# Secondary adrenocortical insufficiency after treatment with retifanlimab: a case report

**DOI:** 10.3389/fimmu.2024.1371527

**Published:** 2024-06-10

**Authors:** Wenjing Zhang, Li Xiao, Guangxin Zhou, Huijuan Zhu, Yongmin Bi, Junjie Du, Da Zhang

**Affiliations:** ^1^ Department of Endocrinology, Air Force Medical Center, Air Force Medical University, Beijing, China; ^2^ China Medical University, Shenyang, China; ^3^ Department of Nuclear Medicine, Air Force Medical Center, Air Force Medical University, Beijing, China; ^4^ Department of Orthopedics, Air Force Medical Center, Air Force Medical University, Beijing, China

**Keywords:** adrenocortical insufficiency, retifanlimab, immune-related endocrine event, immunotherapy, hyponatremia

## Abstract

With advancements in medical oncology, immune checkpoint inhibitors (ICIs) have become the first-line treatment for many malignancies. ICIs play a significant role in improving cancer prognosis, but a series of immune-related adverse events (irAEs), including immune-related endocrine events (irEEs), caused by ICIs have also aroused concerns. Rapid clinical identification of irAEs caused by ICIs is particularly important. We describe a case of secondary adrenocortical insufficiency (AI) after PD-1 treatment in a postoperative patient with endometrial cancer. A 73-year-old female patient developed anorexia, nausea, vomiting, malaise, electrolyte disturbances, ineffective symptomatic treatment, and decreased serum adrenocorticotropin and cortisol levels six months after retifanlimab treatment. The vomiting resolved, and the electrolyte levels were corrected after 3 days of treatment with glucocorticoids (hydrocortisone, intravenous, 200 mg/day). When patients present with gastrointestinal symptoms, such as poor appetite and nausea, not only symptomatic treatment but also a search for the etiology behind the symptoms is needed, especially in immunotherapy patients who should undergo a thorough evaluation of the endocrine system and be alert for adrenocortical insufficiency.

## Introduction

Immune checkpoint inhibitors (ICIs) are monoclonal antibodies that target certain immune checkpoints, such as cytotoxic T lymphocyte-associated protein 4 (CTLA-4), programmed cell death protein 1 (PD-1), and lymphocyte activation gene - 3 (LAG-3), which play a role in promoting T-cell activation and antitumor effects and have become the therapeutic treatment for many malignant tumors (1, 2). ICIs play a significant role in improving the prognosis of cancers, but a series of immune-related adverse events (irAEs) caused by ICIs have also aroused concerns, which develop in about 40–50% of patients (3). ICIs cause irAEs by mechanisms and manifestations that are very different from those of cytotoxic chemotherapy, radiotherapy or molecularly targeted drugs. These irAEs are thought to be autoimmune reactions caused by blockage of normal immune regulation pathways (4, 5) and may affect multiple organ systems. Endocrine diseases, also known as immune-related endocrine events (irEEs), are among an incidence of 8.1% (6). IrEE is characterized by hypophysitis, thyroid dysfunction, insulin-deficient diabetes mellitus, and adrenocortical insufficiency (AI). Retifanlimab is a humanized, hinge-stabilized immunoglobulin (Ig) G4κ monoclonal antibody that recognizes human PD-1 and blocks the binding of programmed cell death-ligand 1 (PD-L1) and programmed cell death-ligand 2 (PD-L2) to PD-1 expressed on the cell surface in a dose-dependent manner (7). As a novel targeted agent, retifanlimab is currently recommended as the first-line agent for perioperative treatment of solid tumors (8). AI has rarely been reported in retifanlimab treatment. We report a case of intractable nausea and vomiting six months after the end of treatment with retifanlimab in a patient with lung metastasis from endometrial cancer, who was finally diagnosed with secondary AI, to increase awareness of rare but serious adverse reactions for early recognition and intervention.

## Case report

A 73-year-old woman who was menopausal at the age of 48 years presented with a 2-year period of irregular vaginal bleeding in August 2017. Pathological examination after curettage indicated moderately to poorly differentiated endometrioid carcinoma with local sarcomatoid differentiation. Computed Tomography (CT) suggested multiple nodular shadows in both lungs, and the possibility of multiple lung metastases was considered. CT guided lung tissue aspiration biopsy was suggestive of adenocarcinoma. Immunohistochemistry: p40(-), CD56(-), CgA(small foci +), Ki-67(90%+), Syn(-), TTF-1(-), PAX8(+) was consistent with lung metastasis of poorly differentiated endometrioid carcinoma. Radical surgery for endometrial carcinoma was performed in September 2017 with removal of the uterus and adnexa. The pathological diagnosis was poorly differentiated endometrioid carcinoma involving deep myometrial invasion (>1/2 of the myometrial thickness), with some areas adjacent to the serosa and tumor emboli visible in the blood vessels. The TNM stage was T_1b_N_0_M_1_, and the FIGO stage was stage IV B. Six cycles of chemotherapy with carboplatin plus paclitaxel followed intravenous dexamethasone were underwent. Chemotherapy was ended in March 2018. Although the lung metastases shrank after treatment, they grew again 2 years post-treatment (details are unknown).

In September 2020, the patient was screened for an MSI-H/dMMR gene mutation (+) and began participating in a clinical trial with retifanlimab. The regimen was planned for 30 cycles, 28 days per cycle, intravenously infusing 500 mg of retifanlimab for 30 min on the first day of each cycle. At the end of the seventh cycle, the patient’s 1 cm patchy erythema in the lower abdomen progressed to widespread erythema throughout the body, accompanied by itching. Psoriasis was diagnosed, and immunotherapy was paused for two cycles. After 2 months of traditional Chinese medicine treatment, itching disappeared, and the erythema on the skin decreased in size. Immunotherapy resumed in May 2021. During the infusion of retifanlimab, the patient had poor appetite, fatigue, and depression; however, the symptoms resolved between treatments. In March 2022, during the 18th cycle of immunotherapy, thyroid function tests revealed the following results: free triiodothyronine (FT3) 8.74 pmol/L (reference range: 3.5–6.5), triiodothyronine (TT3) 3.78 nmol/L (reference range: 0.92–2.79), free tetraiodothyronine (FT4) 11.68 pmol/L (reference range: 7.98-16.02), thyroxine (TT4) 209.00 nmol/L (reference range: 58.10–165.20), thyroid-stimulating hormone (TSH) 0.016 mIU/L (reference range: 0.56–5.91), thyroglobulin antibody (TgAb) 304.7 IU/L (reference range: <4.0), thyroid-stimulating hormone receptor antibody (TRAb) 13.66 IU/L (reference range: <1.75). The patient had no obvious symptoms, such as palpitations, pyrexia, or hand tremors. The patient was diagnosed with Graves’ disease, and methimazole (10 mg/day) was administered orally. On the 28th cycle of immunotherapy, the patient developed erythema with itching all over the body again, and the immunotherapy ended two cycles earlier. Thyroid function was normal, and methimazole was discontinued. Lung CT revealed the disappearance of lung metastases.

After catching a cold in April 2023, the patient experienced poor appetite, nausea, and vomiting. Symptomatic and nutritional support were provided; however, the patient’s symptoms did not improve. On May 4, 2023, laboratory tests revealed potassium level of 2.85 mmol/L and sodium level of 128 mmol/L, respectively. The patient underwent fluid replacement and electrolyte correction; however, no improvement was observed. On May 19, 2023, the patient received emergency medical care.

The patient had a history of hypertension for 10 years, with a maximum blood pressure of 160/110 mmHg. Her blood pressure was maintained at approximately 140/90 mmHg using oral metoprolol 50 mg twice daily. Recently, she had not used any antihypertensive drugs and her blood pressure was normal. She had lost 15 kg of body weight in the previous month.

**Figure 1:**
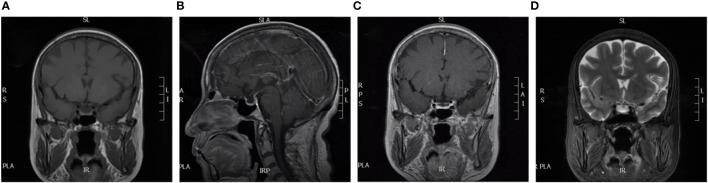
MRI of our patient. **(A)** is coronal; **(B)** is disorientation; **(C)** is coronal dynamic enhancement; **(D)** is coronal T2. All show normal pituitary morphology.

At the time of the patient’s admission, temperature was 36.8°C, oxygen saturation was 98%, and blood pressure was 140/90 mmHg. Clear breath sounds were heard in both the lower lungs, with no adventitious breath sounds. Other physical examinations revealed mild edema of the lower limbs, pubic hair and axillary hair sparsity, no change in skin color, and no dehydration. Laboratory evaluation showed blood potassium 3.73 mmol/L, blood sodium 136.7 mmol/L, blood magnesium 0.54 mmol/L, blood glucose 3.08 mmol/L, blood creatinine 42 umol/L. Further laboratory tests of endocrine function suggested: FT3 5.7 pmol/L, TT3 2.37 nmol/L, TT4 147.54 nmol/L, TSH 3.76 mIU/L, serum cortisol at 8 am 13.54 nmol/L (reference range: 157.81–535.80 nmol/L), adrenocorticotropin (ACTH) 2.81 pg/mL (reference range: 6.0–48 pg/mL), insulin-like growth factor (IGF-1)61.38 ng/mL (reference: 60–350), prolactin (PRL) 720.77 uIU/mL (reference range: Postmenopausal 58.09–416.37 uIU/mL), progesterone(P) 0.42 nmol/L (reference range: Postmenopausal <2.96 nmol/L), estradiol(E2) <55.10 pmol/L, follicle-stimulating hormone(FSH) 20.58 mIU/mL (reference range: Postmenopausal 16.74–113.59 mIU/mL), luteinizing hormone(LH) 11.87 mIU/mL (reference range: menopausal 10.87–58.64 mIU/mL). Magnetic resonance imaging (MRI) of the saddle region revealed no abnormalities (**Figure 1**).

The patient was administered fluid replacement and hydrocortisone succinate 200 mg/day intravenously. Her symptoms resolved and feeding returned to normal. The glucocorticoids were gradually reduced to oral prednisone acetate tablets 5 mg once daily. The patient’s vital signs were stable, and electrolyte levels were normal.

On September 7, 2023, the patient returned to hospital for follow-up. She had no obvious complaints of discomfort, a weight gain of 10 kg, good spirit, scattered red flaky rash visible on the abdominal skin, no full moon face or buffalo back, no purple lines on the skin, clear respiratory sounds in both lungs, or edema in the lower extremities. Blood pressure was 120/70 mmHg without antihypertensive drugs, serum cortisol at 8 am was 15.84 nmol/L, ACTH 3.16 pg/mL, IGF-1 70.00 ng/mL, blood potassium 4.19 mmol/L, blood sodium 145.4 mmol/L, blood glucose 4.53 mmol/L, TT3 1.60 nmol/L, TT4 117.49 nmol/L, FT3 4.44pmol/L, FT4 12.33pmol/L, TSH 3.33mIU/L, TPOAb 0.5IU/L, TgAb<0.9IU/L. In the measurement of sex hormone levels, PRL was 164 uIU/mL, P 0 nmol/L,E2 <55.10 pmol/L, FSH 29.86 mIU/mL, LH 7.45 mIU/mL. Laboratory test results before and after treatment are shown in **Table 1**. No abnormalities were observed on saddle region MRI. The patient was instructed to continue the treatment with oral prednisone (5 mg/day).

**Table 1:** Laboratory tests before and after treatment of the patient.

Inspection items	Pre-treatment	Post-treatment	Reference interval	Unit
Blood potassium	3.73	4.19	3.5–5.3	mmol/L
Blood sodium	136.7	145.40	137–147	mmol/L
Blood magnesium	0.54	0.91	0.75–1.02	mmol/L
Blood glucose	3.08	4.53	3.6–6.1	mmol/L
SCr	42	73	41–81	umol/L
FT3	5.7	4.44	3.53–7.37	pmol/L
TT3	2.37	1.6	1.54–3.08	nmol/L
FT4	11.68	12.33	7.98–16.02	pmol/L
TT4	147.54	117.49	72–136	nmol/L
TSH	3.76	3.33	0.56–5.91	mIU/L
8am Serum Cortisol	13.54	15.84	157.81–535.80	nmol/L
ACTH	2. 81	3.16	6.0–48	pg/mL
IGF-1	61.38	70.00	60–350	ng/mL
PRL	720.77	164.32	58. 09–416.37	uIU/mL
P	0.42	0.00	<2.96	nmol/L
E2	<55.10	<55.10	<142.76	pmol/L
FSH	20.58	29.86	16.74–113.59	mIU/mL
LH	11.87	7.45	10.87–58.64	mIU/mL

SCr, serum creatinine; FT3, free triiodothyronine; TT3, total triiodothyronine; FT4, free tetraiodothyronine; TT4, total thyroxine; TSH, thyroid-stimulating hormone; ACTH, adrenocorticotropic hormone; IGF-1, insulin-like growth factor 1; PRL: Prolactin; P: Progesterone; E2: Estradiol; FSH: Follicle-stimulating hormone; LH, luteinizing hormone.

## Discussion

The patient developed psoriasis and hyperthyroidism during the retifanlimab treatment. Intractable nausea, vomiting, loss of appetite, and fatigue with hyponatremia and hypoglycemia developed six months after the end of treatment, and these symptoms were not relieved by symptomatic treatment. Detailed endocrine evaluation suggested that the patient’s serum cortisol and ACTH levels were low without the use of exogenous glucocorticoids, denying history of infarction, trauma, and surgical resection, pituitary tumors also ruled out with no abnormalities on saddle MRI, supporting immunotherapy-related secondary AI. The PRL levels were mildly elevated because of stress. The patient’s symptoms were resolved with glucocorticoid replacement therapy.

The patient presented to the emergency department with gastrointestinal symptoms, whose clinical presentation and biochemical changes were not specific, especially in cancer patients (9). Electrolyte disturbances due to vomiting can also interfere with diagnosis. This case suggests to the primary care physician that when encountering a patient with a history of immunotherapy who presents with gastrointestinal symptoms and electrolyte disturbances, it is important to consider not only the effects of the tumor and medications, but also irAEs (10). Initial laboratory tests should include basal metabolic tests and cortisol, adrenocorticotropic hormone, TSH and FT4 (11).

According to a meta-analysis of 597 patients receiving immunotherapy, the incidence of AI was 8.5% (12). AI can be categorized as either primary or secondary. Of 206 patients with ICI-induced AI, 92.7% (191) had secondary AI, 5.3% (11) had primary AI, and 1.9% (4) had mixed AI (13). Primary AI results from T cell- and antibody-mediated destruction of the adrenal cortex and is rarer than secondary AI, with a total incidence of only 1.03% (14). Secondary AI can be caused by hypophysitis, which is most often induced by the CTLA-4 inhibitor ipilimumab, with a prevalence of 1.8–17% (13). Hypophysitis has been reported in patients receiving anti-PD-1/PD-L1 therapy, but its incidence is low, 0.5–1% (15). Additionally, patients receiving a combination of anti-CTLA-4 and anti-PD-1 therapies are more likely to develop hypophysitis than those receiving anti-CTLA-4 monotherapy (16). A recent study has shown that combination therapy with LAG-3 and PD-1 inhibitors resulted in a higher incidence of hypophysitis than PD-1 inhibitor monotherapy (2.5% vs. 0.8%, respectively) (2, 3). The pathogenesis of secondary AI is unclear, and it has been suggested that it is related to type II hypersensitivity reactions mediated by IgG or IgM antibodies; there is also evidence of a possible concomitant type IV sensitivity reaction (T-cell-mediated) leading to hypophysitis (16). Hypophysitis induced by PD-1 inhibitor therapy is often an isolated ACTH deficiency, with hyponatremia and gastrointestinal symptoms as the common initial symptoms (17). In hypophysitis, the pituitary gland is enlarged in approximately 12 - 14.3% of patients, stalk only in approximately 21.4 - 22%, and pituitary-stalk is enlarged in 64.3 - 65% (18, 19). PD-1/PD-L1 inhibitor-induced hypophysitis may lack the typical pituitary enlargement and may only present as a secondary AI. In a report of 13 individuals with secondary AI, six (46.2%) developed pituitary enlargement. Five (38.4%) of the seven patients treated with CTLA-4 inhibitor monotherapy or combination therapy developed pituitary enlargement. In contrast, only one (7.6%) of six patients treated with PD-1/PD-L1 inhibitor monotherapy developed an enlarged pituitary gland, and follow-up MRI demonstrated that pituitary enlargement had resolved in all six patients (20). In the present case, the patient developed secondary AI without pituitary abnormalities following retifanlimab administration. In a trial of 43 patients treated with a combination of margetuximab and retifanlimab, 14 (32.6%) experienced nausea, 11 (25.6%) experienced loss of appetite, 11 (25.6%) experienced malaise, and 9 (20.9%) experienced vomiting (21). A literature search did not find any reports of retifanlimab causing secondary AI, which may be related to its short market time; it was first approved in the United States on March 22, 2023 (22).

In addition to pituitary involvement, ICIs can cause adverse reactions in other endocrine glands. Thyroid dysfunction is the most common irEEs (23). The involvement of one endocrine gland may be followed by the involvement of several endocrine glands. Abnormalities in different endocrine glands do not necessarily appear chronologically. Both thyroglobulin- and ACTH-associated immunity are likely to be affected by ICIs treatment, which induces thyroid and ACTH dysfunction (24, 25). ACTH deficiency is often observed in patients with thyroid involvement.

Notably, the patient developed psoriasis during immunotherapy. Skin-related symptoms are common during immunotherapy. A clinical diagnosis of immune-related lichen planus has been reported in patients who developed skin, oral, and genital mucosal involvement after the second cycle of retifanlimab (26). When skin toxicity occurs, thyroid function and creatinine levels need to be monitored more closely because thyroid inflammation and nephrotoxicity are more common in patients presenting with adverse skin reactions (27). No studies have clearly indicated a relationship between the skin and adrenal insufficiency. Systemic stress mediators, including cortisol and ACTH, can induce various cutaneous immune and inflammatory responses that affect skin cells/appendages and their targets (28). Adverse skin effects of immunotherapy may be accompanied by irEEs.

The onset of irEEs varies across ICIs treatments, with most occurring within 5 months of treatment initiation and a few occurring after ICIs cessation (4). Anti-CTLA-4 monotherapy is usually performed within 12 weeks, whereas anti-PD-1 monotherapy has an onset time of 0–48 weeks (29). The median times to primary and secondary AI after initiation of ICIs therapy were 200.5 (35–280) and 178 (16–562) days, respectively (30). We are reminded that even after the end of immunotherapy, attention should be paid to irAEs, and endocrine-related problems should be detected promptly.

There are some limitations. Firstly, the patient did not undergo endocrine evaluation before chemotherapy and immunotherapy, ignoring the possible side effects of the treatment. We also recommend a baseline endocrine evaluation before starting chemotherapy and immunotherapy. Secondly, the patient did not undergo insulin tolerance test or growth hormone-releasing hormone plus arginine stimulation test. The patient was admitted to the hospital with electrolyte disturbances, we considered the presence of AI. Considering the risks associated with the tests in this condition, as well as the patient’s IGF-1 levels being above the lower limit of normal range at that time, we did not conduct stimulation tests to identify growth hormone deficiency. We will monitor for growth hormone deficiency during long-term follow-up. In addition, according to the clinical practice guidelines of the Endocrine Society, treatment for patients with AI includes the use of hydrocortisone or cortisone acetate, both of which are physiological substitutes for glucocorticoids (31). However, owing to issues such as drug procurement, hydrocortisone or cortisone acetate is not available in our hospital. Therefore, prednisone was used as an alternative treatment option after comprehensive consideration.

## Conclusion

The effects of ICIs persist over a long period of time. Although fewer side effects have been reported for retifanlimab, in the light of experience with other immunosuppressive drugs, clinicians should be highly suspicious of irEEs in patients who develop malaise, gastrointestinal symptoms, electrolyte disturbances, or a history of previous endocrine gland involvement after ICIs treatment, whether they are in the middle of immunotherapy or at the end of treatment, especially in patients with unexplained electrolyte abnormalities. Long-term screening for endocrine function should be continued even after the discontinuation of immunotherapy.

## Data availability statement

The original contributions presented in the study are included in the article/supplementary material. Further inquiries can be directed to the corresponding author.

## Ethics statement

The studies involving humans were approved by Air Force Medical Center Ethics Committee. The studies were conducted in accordance with the local legislation and institutional requirements. The participants provided their written informed consent to participate in this study. Written informed consent was obtained from the individual(s) for the publication of any potentially identifiable images or data included in this article.

## Author contributions

WZ: Writing – original draft, Writing – review & editing. LX: Writing – review & editing. GZ: Writing – review & editing. HZ: Writing – review & editing. YB: Writing – review & editing. JD: Writing – review & editing. DZ: Writing – original draft, Writing – review & editing.
